# Seidlmayer’s purpura: five cases and review of the litterature

**DOI:** 10.1186/1546-0096-12-S1-P360

**Published:** 2014-09-17

**Authors:** Maria Cristina Maggio, Eugenia Prinzi, Laura Triolo, Giovanni Corsello

**Affiliations:** 1 Pro.Sa.M.I. "G. D'Alessandro", University of Palermo, Palermo, Italy

## Introduction

About 100 cases of AHEI have been published in medical literature worldwide. Although initially considered a variant of Henoch-Schönlein purpura (HSP), it is now considered a separate entity: in fact it shows infrequently visceral involvement and IgA skin depositions. Furthermore these patients show a better prognosis than HSP patients. Onset age for AHEI usually ranges between 4 and 24 months but it spreads from birth to 60 months. AHEI, also defined Seidlmayer’s purpura (SP), is characterized by the triad: fever, oedema and purpura. The latter is usually rosette-, annular- or targeted-shaped primarily over the face, ears and extremities in a nontoxic infant. The development and the rapidity of the skin lesions’ onset are typical and more frequent in winter. Skin lesions are dramatic both in appearance and rapidity of onset. In some cases viral or bacterial infections, drugs, vaccinations are documented.

## Objectives

However AHEI is a self-limited short-duration disease, usually lasting less than 3 weeks. Long-term sequelae are unlikely and relapses are uncommon.

## Methods

We report five cases of SP, age: 8-11 months, admitted since January 2013 till April 2014. The purpuric lesions were localized on the face, on the distal ends of limbs on gluteal region. Leucocytes, CRP, transaminases, urine, BUN, creatinin were in the normal ranges. The good clinical conditions of the children allowed excluding a septic framework. The abdomen scan excluded the presence of intestinal loops thickening, suggestive of an involvement of small vessel district. A dermatological evaluation confirmed the suspicion of SP. Serological test of IgM and IgG for Epstein-Barr, Parvovirus, Adenovirus, Coxackie, Mycoplasma Pneumoniae, Chlamydia did not confirm a recent infection. Anti-nuclear antibodies (ANA) and antineutrophil cytoplasmatic antibodies (ANCA) were undetectable.

A nasopharyngeal swab was negative for pathogen bacteria and virus as Influenza, Parainfluenza, Respiratory Syncytial virus, Beta-hemolytic group A Streptococcus in 3, positive for Parainfluenza virus in 1, Haemophilus Influentiae in 1. in 2 patients skin lesions were detected 1-2 days after amoxicillina plus clavulanic acid were started.

## Results

In 3 patients the fecal occult blood was positive; in 2 fecal occult blood and the urine examination were negative during the acute phase of the illness and the follow up.

The patients with the fecal occult blood positive, skin lesions had an ulcerative evolution in the fingers, toes and/or the ears. The worsening of skin lesions suggested the treatment with methylprednisolone (1mg/kg/die with benefit, tapering on the fith-sixth day and completely suspended after 7-8 days).

## Conclusion

In most cases no specific treatment is required for SP. The role of corticosteroids is limited to forms with intestinal involvement. In 3of our patients corticosteroids were necessary due to the severity of dermatological lesions, with a successful improvement in the clinical outcome. Recognition of this rare purpura will avoid misdiagnosis, anxiety for parents and inappropriate therapeutic approaches.

## Disclosure of interest

None declared

